# Efficacy of pulmonary rehabilitation in patients with moderate chronic obstructive pulmonary disease: a randomized controlled trial

**DOI:** 10.1186/1471-2296-14-21

**Published:** 2013-02-11

**Authors:** Miguel Román, Concepción Larraz, Amalia Gómez, Joana Ripoll, Isabel Mir, Eduardo Z Miranda, Ana Macho, Vicenç Thomas, Magdalena Esteva

**Affiliations:** 1 Primary Care Majorca Department, Son Pisà Primary Health Centre, C/ Vicenç Joan Perello Ribes, 65, Palma de Mallorca, Baleares, Spain; 2 Primary Care Majorca Department, Escuela Graduada Primary Health Centre, Palma de Mallorca, Baleares, Spain; 3 Primary Care Majorca Department, Coll d´en Rabassa Primary Health Centre, Palma de Mallorca, Baleares, Spain; 4 Primary Care Majorca Department, Unit of Research, Palma de Mallorca, Baleares, Spain; 5 Primary Care Majorca Department, Hospital Son Llàtzer, Unit of Pneumology, Palma de Mallorca, Baleares, Spain; 6 Primary Care Majorca, Emili Darder Primary Health Centre, Palma de Mallorca, Baleares, Spain; 7 Primary Care Madrid Department, Aspes Primary Health Centre, Madrid, Spain; 8 Primary Care Majorca, Camp Redó Primary Health Centre, Palma de Mallorca, Baleares, Spain

**Keywords:** Chronic obstructive pulmonary disease, Pulmonary rehabilitation, Quality of life, Clinical trial, Primary care

## Abstract

**Background:**

Pulmonary Rehabilitation for moderate Chronic Obstructive Pulmonary Disease in primary care could improve patients’ quality of life.

**Methods:**

This study aimed to assess the efficacy of a 3-month Pulmonary Rehabilitation (PR) program with a further 9 months of maintenance (RHBM group) compared with both PR for 3 months without further maintenance (RHB group) and usual care in improving the quality of life of patients with moderate COPD.

We conducted a parallel-group, randomized clinical trial in Majorca primary health care in which 97 patients with moderate COPD were assigned to the 3 groups. Health outcomes were quality of life, exercise capacity, pulmonary function and exacerbations.

**Results:**

We found statistically and clinically significant differences in the three groups at 3 months in the emotion dimension (0.53; 95%CI0.06-1.01) in the usual care group, (0.72; 95%CI0.26-1.18) the RHB group (0.87; 95%CI 0.44-1.30) and the RHBM group as well as in fatigue (0.47; 95%CI 0.17-0.78) in the RHBM group. After 1 year, these differences favored the long-term rehabilitation group in the domains of fatigue (0.56; 95%CI 0.22-0.91), mastery (0.79; 95%CI 0.03-1.55) and emotion (0.75; 95%CI 0.17-1.33). Between-group analysis only showed statistically and clinically significant differences between the RHB group and control group in the dyspnea dimension (0.79 95%CI 0.05-1.52). No differences were found for exacerbations, pulmonary function or exercise capacity.

**Conclusions:**

We found that patients with moderate COPD and low level of impairment did not show meaningful changes in QoL, exercise tolerance, pulmonary function or exacerbation after a one-year, community based rehabilitation program. However, long-term improvements in the emotional, fatigue and mastery dimensions (within intervention groups) were identified.

**Trial registration:**

ISRCTN94514482

## Background

Chronic obstructive pulmonary disease (COPD) is a major, worldwide health problem. In Spain, COPD affects 10.2% of individuals aged 40–80 years and is the fourth leading cause of death [1]. COPD is a progressive disease, with chronic and worsening air-flow limitation. In addition to pathological changes in the lungs, COPD has been associated with extra-pulmonary manifestations which contribute to disease severity [2]. The only therapeutic measures that have proven effective in avoiding disease progression are smoking cessation and continuous home-based oxygen therapy in patients with hypoxemia [3,4]. Other treatments are associated with control of symptoms, such as improving dyspnea and patient quality of life, and avoiding exacerbations and hospitalizations.

Pulmonary rehabilitation (PR) is an evidence-based, multidisciplinary, comprehensive intervention for patients with chronic respiratory diseases who are symptomatic and often have progressively restricted daily-life activities. Pulmonary rehabilitation should be considered for all patients with chronic respiratory disease who have persistent symptoms, limited activity, and/or are unable to adjust to the illness despite otherwise optimal medical management. Gains can be achieved from pulmonary rehabilitation regardless of age, sex, lung function, or smoking status [5]. This comprehensive intervention has been clearly demonstrated to reduce dyspnea, improve exercise performance and health-related quality of life (HRQL), and is a significant component in the management of COPD [6]. Furthermore, an emerging literature is beginning to reveal its effectiveness in reducing health-care costs [7]. PR reduces hospital admissions and mortality compared with usual community care (no rehabilitation) in COPD patients following an exacerbation and appears to be a highly effective and safe intervention. In spite of these clear benefits, only a small proportion of COPD patients have access to PR programs. Data from Canada indicates that less than 1.2% of patients with COPD had access to a PR program per year [8].

PR can be delivered in a variety of structured programs that may themselves have an influence on the degree or duration of long-term benefit [5]. Principles of pulmonary rehabilitation apply regardless of location and it has been shown to be effective across various settings including outpatient and home-care [9-13]. Pulmonary rehabilitation delivered in a community setting has similar efficacy to that achieved in a more traditional hospital-based setting. The potential advantages of community PR programs include cost-effectiveness, a safe clinical environment and the availability of trained staff [5]. Some studies show longer-lasting benefits derived from home-based rather than hospital-based programs [14] along with evidence that they provide greater patient comfort and satisfaction [15].

Few studies of a multidisciplinary nature have been performed in the Primary Health Care setting. One integrated disease-management program (consisting of optimal medication, reactivation, education, and exacerbation management) was developed for primary care patients with COPD in the Netherlands resulting in improved QoL and decreased dyspnea one year after program completion [16]. Another nurse-led, multidisciplinary pulmonary rehabilitation program in primary health care in Sweden found no significant improvement in functional capacity and QoL but did find a reduction in exacerbations at 12 months following a 6-week program [17]. The choice of model will depend on local factors of convenience, existing availability of resources and incremental costs. Staff characteristics may be important in achieving optimal outcomes [18].

Other factors associated with PR programs remain uncertain, including the long-term preservation of benefits and their optimal duration. Various strategies have been tried to maintain the benefits of rehabilitation [11,14,15]. Continuing rehabilitation for a prolonged period only seems to confer a small additional benefit and comparisons between 18-month and 3-months programs have not provided greater insight into the matter [19]. There have been other studies of specific maintenance interventions after a conventional course of rehabilitation but, as yet, there is no broad consensus as to their benefit [5].

We compared the efficacy of a 3-month PR program (education and supervised training) with or without further PR maintenance over 9 months in primary health care centers with that of usual care, with the aim of improving the quality of life of patients with moderate COPD.

## Methods

### Design

This study is a parallel-group, randomized clinical trial involving 3 groups of patients with moderate COPD. The first group received PR for 3 months and rehabilitation maintenance for 12 months (RHBM group). The second group received PR for 3 months without further maintenance (RHB group) and the third (control) group received routine care without rehabilitation (GC group). The study protocol has been published elsewhere [20].

### Selection of patients

Patients were recruited by their family physician from 7 primary care practices in Palma de Mallorca, Spain, from July 2005 to March 2007. Patients were identified from both the health center COPD registry and from the results of spirometry performed at the participating health centers. Subjects were eligible if they were 35 to 74 years old, had moderate COPD according to the GOLD criteria and had post-bronchodilator results on most recent spirometry of FEV1/FVC <0.7, FEV1 values between 50% and 80% [2], and were either smokers or non-smokers. Subjects were excluded if they presented with any musculoskeletal conditions that prevented exercising and walking test assessments, or terminal illness or other severe disease at the time of enrollment. Identified patients attended an inclusion visit with a family physician researcher to check eligibility. During this visit they were asked for written informed consent prior to randomization and baseline measurements. Patient recruitment lasted 12 months.

### Intervention

Patients in the two intervention groups participated in a PR program, delivered in two primary care health centers, consisting of three 60-minute sessions each week for 3 months. The intervention involved groups of 5–10 patients. The sessions consisted of three types of interventions:

a) Education program. During weeks 1, 6, and 12, patients received a 45-minute education session on the anatomy and physiology of the respiratory system, the correct use of inhalers and brief counseling on smoking cessation.

b) Respiratory Physiotherapy. Each session included a series of exercises, lasting a total of 15 minutes and including self-conscious breathing control, diaphragmatic breathing control, and exercises for the chest wall and abdominal muscle walls.

c) Low intensity peripheral muscle training. Each session included abdominal and upper and lower limb exercises, shoulder and full arm circling, weight-lifting and other exercises. This training has been described previously [21] and used in other clinical trials [22,23]. Each exercise was repeated 8–10 times over 45 minutes.

Programmed activities were supervised by two physiotherapists in two primary health centers. They were trained for 7 days in a hospital PR unit in Barcelona. The physiotherapist recorded patient attendance at each session.

After completing the 3-month PR program, patients in the RHBM group attended a weekly-session maintenance program, including both respiratory physiotherapy and low intensity peripheral muscle training, until the end of the program at 12 months. Patients in the RHB group participated in routine care with their physician and nurse.

### Control group

These patients did not participate in either of the intervention programs; rather, they remained under the routine care of their general practitioner and nurse throughout.

### Randomization & blinding

After the inclusion visit and before baseline evaluation visit, subjects were randomly assigned to one of the three groups using a centrally administered, computer-generated block randomization scheme using blocks of 6 with EPIDAT, stratified according to participating site. The case manager subsequently informed patients of their group allocation at the end of the baseline visit. The baseline visit was scheduled when enough patients were recruited to begin a PR group. Health staff members involved in follow-up (a psychologist and a nurse) were blinded to patient assignment.

### Measurements

Evaluation visits were scheduled at enrollment, baseline, and after 3 and 12 months by the study secretary. At each contact, reasons for not attending PR sessions and evaluation were collected. During the assessment visits, the Chronic Respiratory Questionnaire was completed by a psychologist. Subsequently, patients responded to the rest of the questionnaire and underwent the 6-minute walking test (6MWT) and spirometry under nurse supervision.

### Primary outcome variable

The pre-specified primary outcome was the change in score on the Spanish validated version of the Chronic Respiratory Questionnaire (CRQ) [24]. Each of the 20 items, in 4 domains (dyspnea, fatigue, emotional function and mastery or level of control), was graded on a seven-point Likert scale. An average change in score per item of 0.5 represented minimal, clinically relevant improvement or worsening [25].

### Secondary outcome variables

*Pulmonary function tests* included forced spirometry and reversibility test with salbutamol. According to ERS-ATS recommendations, we used a Datospir 120 spirometer and measured FVC, FEV1 and FEV1/FVC, with results compared to Roca-Separ Spanish-specific reference values [26].

*Exercise tolerance* was assessed through the 6MWT. Two tests were performed per session, with a rest of at least 30 minutes between tests and the best result was used for each patient [27]. During the test, oxygen saturation and heart rate were monitored using a portable saturation monitor. Tests were stopped if subjects experienced chest pain, mental confusion, intolerable dyspnea or oxygen saturation <90. The Borg dyspnea scale was completed before and after each 6MWT.

*Any hospital admissions and visits to family physician due to COPD exacerbations. Exacerbation* was defined as episodes of either dyspnea or dry or productive cough, whether sputum was purulent or not, which were treated with oral corticosteroids and/or antibiotics. Also treatments with antibiotics or corticosteroids. These variables were recorded at baseline and final visit. At the baseline visit, patients were asked about episodes during the 12 months before enrollment and, at the final visit, about episodes suffered during follow-up. This information was confirmed by a nurse researcher review of primary care and hospital medical records.

Others variables included group allocation; demographic variables such as sex, age, smoking habits and packs/year; treatments for COPD; and dyspnea, as measured by the Medical Research Council (MRC) [28].

### Sample size

Sample size calculation was based on the difference in expected improvement in quality of life between the intervention RHBM group and the control group. This improvement was defined as the difference between the mean global score on the CRQ scale at baseline and post-rehabilitation. Sample size was calculated with ENE program version 2.1, Glaxo SA. A difference of 10 (0.5 per item for 20 items) was recognized as the minimum, clinically relevant difference. With an α level of 0.05, a 1-beta of 0.8 and a standard deviation of 17 [22], and anticipating a drop-out rate of 20%, we planned to randomly assigned 56 subjects to each group.

### Statistical analysis

All statistical analyses were carried out on a modified intention-to-treat basis with SPSS v.14. Some patients had difficulty in keeping evaluation appointments. Missing values at evaluation visits were substituted with basal values. However, the numbers of hospital admissions and exacerbations, and the usual treatment of patients, were not replaced by basal values and were scored as missing values.

To reduce bias caused by large withdrawal rate, we did sensitivity analysis. We performed per-protocol analysis, analysis with baseline values for missing data and analysis with multiple imputation for missing data. The three analyses gave the same results. Reported results are for analysis with baseline values replacing missing data.

The chi-square test was used to compare between-group smoking habits and health services utilization for COPD exacerbation. Comparisons of normal variables among the three groups were assessed by ANOVA together with the Tukey post-hoc test.

For the primary outcomes (Chronic Respiratory Questionnaire subscale score, pulmonary function and 6-minute walking) we calculated within-group differences from baseline and 95%CI (with a fixed-effects regression model) adjusting for baseline scores using the treatment group as a predictor. Separate regression analyses predicted treatment differences at 3-month and 12-month evaluations. We conducted generalized linear models analyses (GLM) to estimate adjusted treatment differences and within-group and between-group differences, and the Bonferroni test to compare between-group differences. For the secondary outcomes, we used Poisson regression to analyze exacerbation rates requiring hospitalization and negative binomial to estimate the other variables covering healthcare utilization data. All the analysis was two-tailed, with an alpha level of 5%.

The study received the approval of the Baleares Ethics Committee and the Majorca Primary Care Research Committee.

## Results

We identified 97 patients who met all inclusion criteria and none of the exclusion criteria. All agreed to participate. Allocation resulted in 32 subjects randomized to the control group, 33 to the RHB group and 32 to the RHBM group. For some patients, the lag time from enrollment to basal evaluation was longer than expected because basal evaluations did not start until a minimum number of subjects were gathered for a rehabilitation group. The mean interval-time for each group was: 87 days for the control group, 110 days for the RHB group and 94 days for the RHBM group (Table 1). No significant between-group differences were found.

**Table 1:** Characteristics of study participants at baseline

	**Control n (%)**	**RHB n (%)**	**RHBM n (%)**
Number of subjects	23 (32.4)	22 (31)	26 (36.6)
*Sex*			
Female	4 (17.4)	4 (18.2)	5 (19.2)
Male	19 (82.6)	18 (81.8)	21 (80.8)
*Working*			
Yes	5 (22.7)	6 (27.3)	7 (26.9)
*Smoking habits*			
Smoker	8 (34.8)	7 (31.8)	9 (34.6)
Never smoked	2 (8.7)	0 (0.0)	1 (3.8)
Ex-smoker	13 (56.5)	15 (68.2)	16 (61.5)
*MRCs dyspnea score category*			
0	11 (47.8)	7 (31.8)	4 (15.4)
1	11 (47.8)	11 (50.0)	17 (65.4)
2	1 (4.3)	2 (9.1)	5 (19.2)
3	0 (0.0)	1 (4.5)	0 (0.0)
4	0 (0.0)	1 (4.5)	0 (0.0)
Lag time enrolment to basal/ evaluation (days), *mean (SD)*	87.4 (85.1)	110.1 (103.2)	93.8 (89.5)
Age *(years), mean (95%CI)*	63.4 (60.4-66.4)	64.1 (59.9-68.2)	64.9 (62.1-67.7)
Body Mass Index, *mean (95%CI)*	28.4 (26.3-30.5)	27.6 (25.4-29.9)	29.8 (27.8-31.7)
*CRQ, mean (95%CI)*			
Fatigue	5.7 (5.2-6.2)	5.3 (4.8-5.7)	5.2 (4.6-5.8)
Mastery	6.3 (5.7-7.0)	7.2 (6.3-8.1)	7.2 (6.2-8.2)
Dyspnea	2.5 (1.8-3.1)	3.0 (2.5-3.6)	3.0 (2.4-3.5)
Emotion	5.2 (4.6-5.8)	4.6 (4.0-5.3)	4.2 (3.6-4.8)
Pulmonary function *mean (95%CI)*			
FVC (l)	74.0 (66.5-81.5)	73.7 (66.4-81.0)	73.4 (67.2-79.6)
FEV1 (l)	60.1 (55.6-64.4)	59.9 (54.9-64.8)	60.9 (56.3-65.5)
FEV1/FVC	59.1 (54.5-63.1)	60.8 (56.5-65.1)	61.2 (58.6-63.8)
Walking test (m), *mean (95%CI)*	436.2 (402.8-469.6)	466.7 (432.0-501.5)	450.7 (413.4 -488.1)
COPD exacerbation hospitalization			
*Yes n (%)*	5 (21.7)	5 (22.7)	3 (11.5)
*mean (95%CI)*	0.6 (0.01-1.2)	0.8 (−0.1-1.8)	1.2 (0.21-2.19)
COPD exacerbation visit to family physician			
Yes n (%)	12 (52.2)	9 (40.9)	7 (28)
*mean (95%CI)*	3 (−2.4-8.4)	0.5 (−0.01-0.9)	0.5 (−0.04-0.9)
COPD treatment with antibiotics or corticoids			
Yes n (%)	11 (50)	8 (36.4)	9 (36)
*mean (95%CI)*	0.6 (0.01-1.2)	0.8 (−0.1-1.7)	1.2 (0.2-2.2)

l = litres; m = meters; CI = Confidence Interval; χ^2^ for categorical variables and ANOVA for continuous variables.

No significant between-group differences were found for any of the variables.

**Figure 1:**
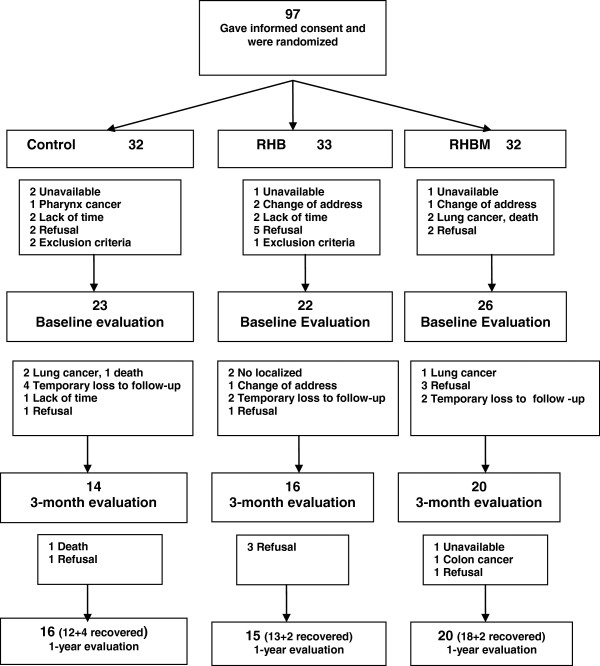
Study design.

During that period some patients were lost and did not attend basal evaluation. Finally, 23 patients were included in the control group, 22 in the RHB group and 26 in the RHBM group; these patients were included in our analysis. Figure 1 summarizes the patient flow and follow-up evaluation. Some subjects withdrew between randomization and baseline evaluation and others were temporarily lost to follow-up, but were evaluated after 12 months. Subjects in the RHBM group attended an average of 25 sessions each, or 69.4% of the planned sessions during the first three months, and an average of 21 during the maintenance period, that is, 58.3% of the planned sessions. Subjects in the RHB group attended an average of 13 sessions, or 36.1% of the sessions over the 3 month period.

Patients’ socio-demographic characteristics included in the analysis showed similar distributions among the three groups at baseline, with none of the parameters showing statistically significant differences (Table 1). Of the patients who withdrew, 82.5% were men with an average age of 62.1 (SD = 15.9) years and a FEV1 of 60% (SD = 10.6). Disease severity and functional capacity, as well as CRQ scores, were also well balanced among the distinct intervention groups.

### Primary outcome

Within-group comparison (Table 2) showed significant and clinically relevant improvements in CRQ emotion scores at 3 months in the two rehabilitation groups. These effects were maintained at 12 months in the RHBM group. Also, significant and clinically relevant improvements were observed in the RHBM group at 3 and 12 month follow-up for fatigue. However, in the mastery dimension, the improvement only reached significant and clinically relevant differences at 12 months in the RHBM group. Between-group comparisons included in Table 3 show significant differences between the control and RHM group at 3 months for dyspnea scores. Between-group changes for other CRQ dimensions scores were not significant and clinically unimportant. Per protocol analysis and multiple imputation of missing values generated the same results.

**Table 2:** Chronic respiratory questionnaire subscale score, pulmonary function and 6-minute walking test differences from baseline to 3 and 12 months

	**Control n = 23**		**RHB n = 22**		**RHBM n = 26**	
	3 months	12 months	3 months	12 months	3 months	12 months
CRQ						
Fatigue	0.4 (−0.05-0.7)	0.2 (−0.1-0.6)	0.03 (−0.3-0.4)	0.3 (−0.1-0.6)	0.5 (0.2-0.8)*	0.56 (0.2-0.9)*
Mastery	−0.6 (−1.1- - 0.1)*	0.3 (−0.5-1.2)	−1.01 (−1.5- -0.5)*	0.01 (−0.8-0.8)	−0.3 (−0.8-0.15)	0.79 (0.03-1.5)*
Dyspnea	−0.5(−0.9- - 0.1)*	−0.4 (−0. 8–0.01)	0.3 (−0.1-0.7)	0.07 (−0.3-0.5)	−0.2 (−0.6-0.2)	−0.23 (−0.6-0.1)
Emotion	0.5 (0.06-1.01)*	0.00 (−0.6-0.6)	0.7 (0. 3–1.2)*	0.5 (−0.13-1.11)	0.9 (0.5-1.3)*	0.7 (0.2-1.3)*
FVC (l)	−1.5 (−4.7-1.7)	1.42 (−2.4-5.3)	0.5 (−2.8-3.9)	3.0 (−1.04-7.05)	−2.4 (−5.5-0.6)	−0.9 (−4.7-2.7)
FEV1 (l)	0.8 (−2.5-4.2)	−0.22 (−1.3-0.9)	−0.03 (−3.5-3.5)	−0.1 (−1.3-0.9)	−2.1 (−5.3-1.1)	−1.2 (−2.2- - 0.1)*
*FEV1/FVC*	0.8 (−1.9-3.6)	3.03 (0.6-5.5)*	−0.5 (−3.3-2.2)	−1.8 (−4.3-0.6)	−0.3 (−2.8-2.2)	−0.08 (−2.3-2.1)
Walking test (m)	27.3 (4.4-50.3)*	33.2 (8.2-58.3)*	0.9 (−22.6-24.3)	20.2 (−5.3-45.8)	19.5 (−2.07-41.1)	19.7 (−3.8-43.3)

Within-group differences from baseline (95%CI).

*P < 0.05. Values are means and 95%CI’s. A difference greater than 0.5 (improvement) or greater than −0.5 ( deterioration) in CRQ scores, is considered clinically important.

m = meters.

**Table 3:** Chronic respiratory questionnaire subscale score, pulmonary function and 6-minute walking test differences from baseline to 3 and 12 months

	3 months			12 months		
	RHB-Control n = 23	RHBM-Control n = 22	RHBM-RHB n = 26	RHB-Control n = 23	RHBM-Control n = 22	RHBM-RHB n = 26
CRQ						
Fatigue	−0.3 (−0.9-0.2)	0.09 (−0.5-0.6)	0.4 (−0.1-0.9)	0.02 (−0.6-0.6)	0.3 (−0.3-0.9)	0.3 (−0.3-0.9)
Mastery	−0.4 (−1.3-0.5)	0.3 (−0.5-1.1)	0.7 (−0.1-1.5)	−0.3 (−1.8-1.1)	0.4 (−0.9-1.8)	0.8 (−0.6-2.1)
Dyspnea	0.8 (0.05-1.5)*	0.3 (−0.4-0.9)	−0.5 (−1.2-0.2)	0.5 (−0.2-1.1)	0.1 (−0.5-0.8)	−0.3 (−0.9-0.3)
Emotion	0.2 (−0.6-0.9)	0.3 (−0.5-1.1)	0.1 (−0.6-0.9)	0.5 (−0.6-1.6)	0.7 (−0.3-1,8)	0.3 (−0.8-1.3)
FVC (l)	2.05 (−3.7-7.8)	−0.9 (−6.4-4.5)	−2.9 (−8.6-2.6)	1.6 (−5.2-8.4)	−2.4 (−8.9-4.1)	−3.9 (−10.7-2.7)
FEV1 (l)	−0.9 (−6.8-5.04)	−2.9 (−8.6-2.7)	−2.05 (−7.8-3.7)	0.07 (−1.8-1.9)	−0.9 (−2.8-0.9)	−1.04 (−2.9-0.8)
*FEV1/FVC*	−1.4 (−6.1-3.4)	−1.12 (−5.7-3.4)	0.2 (−4.3-4.8)	−4.8 (−9.1- -0.6)*	−3.1 (−7.2-0.9)	1.7 (−2.3-5.8)
Walking test (m)	−26.4 (−66.5-13.6)	−7.8 (−46.3-30.7)	18.6 (−20.3-57.8)	−12.9 (−56.7-30.7)	−13.5 (−55.5-28.5)	−0.5 (−43.02-41.9)

*P < 0.05. Values are means and 95%CI’s. A difference greater than 0.5 (improvement) or greater than −0.5 (deterioration) in CRQ scores, is considered clinically important.

m = meters.

Between-group differences (RHB group minus control group, RHBM minus control, RHBM-RHB).

### Secondary outcomes

All 3 groups maintained pulmonary function, with no relevant significant changes, at both time points (Table 2 and Table 3). No differences within or between groups were observed in the 6-MWT. As shown in Table 4, no statistically significant differences were noted in health care outcomes.

**Table 4:** Use of health services due to COPD exacerbations at 12 months

	**12-month evaluation**		
	**Control**	**RHB**	**RHBM**
COPD exacerbation hospitalization			
Yes n (%)	3 (15.8)	5 (22.7)	3 (12.5)
*mean (CI 95%)*	0.2 (−0.05-0.5)	0.2 (−0.04-0.4)	0.1 (−0.02-0.3)
COPD exacerbation visit to family physician			
Yes n (%)	9 (42.8)	7 (35)	7 (30.3)
*mean (CI 95%)*	0.8 (0.1-1.4)	0.7 (0.1-1.4)	0.8 (0.2-1.4)
COPD treatment with antibiotics or corticoids			
Yes n (%)	6 (33.4)	5 (26.3)	4 (20)
*mean (CI 95%)*	1.08 (0.2-1.9)	0.3 (−0.04-0.7)	0.7 (0.1-1.2)

No significant between-group differences were found for any of the variables.

## Discussion

Our study evaluated the long-term effects of a PR program in a community primary care setting with moderate COPD patients. All the groups that took part in PR showed a significant improvement in the emotional dimension after 3 months. This improvement was only maintained in the group that completed the one-year maintenance PR program but not in the 3-month intervention group. Benefits in the fatigue and mastery dimensions also observed after one year of program maintenance were not achieved in the other 2 comparison groups. Lack of improvement in exercise tolerance and overall QoL ratings between groups did not allow accurate determination of PR short- and long-term effectiveness.

Previous studies reported that emotional improvement after the intervention was not maintained after discontinuation or not even after complete PR maintenance programs [29,30], being the first deteriorated QoL dimension. Our results are in line with those reported by Moullec and Chavannes where PR maintenance strategies carried out in the community were associated with long-term improvements in QoL [16,31]. Emotional benefits were observed in both PR groups at 3 months and persisted in the group that continued pulmonary rehabilitation together with improvements in two domains that include socio-psychological components. One explanation could be the beneficial results of mixing with individuals with similar problems and sharing negative experiences with disease. The social support of other COPD patients and the long-term attention of a physiotherapist would promote a reduction in emotional reactions and development of adapted behaviors [31].

Otherwise, between-group findings are not in line with the results of Lacasse et al. meta-analysis [6], as we were not able to find a consistent improvement in any quality-of-life dimension. The patient’s QoL was generally good at baseline and therefore had little scope to show large improvements through low intensity PR activities. Only dyspnea improved significantly after 3 months of PR in the RHB group compared to controls. This results could appear paradoxical given the very low attendance rate (36%) in this group; much lower than the RHBM group (69%). Patients showed a low baseline level of dyspnea in the MRC scale and high exercise capacity in the 6MWT. In fact, 22 patients had dyspnea with intense exercise (MRC 0); of the 47 patients with higher scores, only 17% had a MRC scale score >1.

In common with most PR studies conducted on COPD patients, we found no improvements in pulmonary function parameters [10,21,32]. Our patients following the rehabilitation program did not show significant improvements in exercise capacity in contrast to those described in a previous meta-analysis [6], and in a more recent study [33] where PR, including a much more intensive exercise such as endurance training, was highly effective in improving the exercise capacity of patients with COPD. Our RHB program included low intensity peripheral muscle training but did not have any endurance training which could explain this lack of effect. As mentioned above, we included patients with a high exercise capacity with a mean walking distance between 436 and 466 meters while the Italian study baseline mean distance was around 300 meters [33].

By selecting patients with low basal symptoms, the effectiveness of the intervention can be limited as described by others [17]. These findings suggest that the greatest room for improvement in primary care patients can be expected in those with tangible dyspnea and impaired health status (MRC score > 2 and/or CCQ score >1) across all GOLD stages [16,34].

We found that the proportions of individuals with some exacerbation episodes were similar in the three groups, in agreement with previous studies [35,36], while a recent PR primary care program found a reduction in exacerbation rate after a community PR program [17].

Most PR programs are based at hospitals or at home [10-12,14,33,37]. The intervention we evaluated was developed in primary care centers, with the existing resources of those health centers. In so doing, we attempted to show that this intervention was feasible in a setting where PR is not a regular service but highly accessible for patients. Few PR integrated community programs have been developed with primary care resources and though they found benefits, the results are not conclusive [16,17]. Low patient compliance with the intervention indicates the need for a more integrated approach in order to involve patients as active partners in their treatment process and achieve positive results in behavior modification [31,38].

Our study showed persistent within-group improvements in QoL after only a 1-year maintenance program, with no similar effects in the 3-month program group. At this moment it is not known how long these programs should take and how they might influence behavior [38] or whether alternative management such as an action-plan or self-management strategies could result in important long-term benefits [32,39]. It is generally believed that longer programs yield more durable training effects but the majority of existing studies looking for long-term results, such as ours, have shown small improvements which may not always be possible to replicate [11,15-17,19,23,32,34].

### Limitations

One important limitation arises from the fact that we were not able to recruit the sample size required. Even with active review of included-centers’ registers and COPD patients records, fewer patients than expected were identified. This is consistent with the results of other studies showing COPD under-reporting [40] and the difficulties of recruiting patients with COPD to a PR trial. This affected the power of the study to identify significant changes in the PR groups and led to negative results.

The high rate of withdrawal was also an obstacle to obtaining fair results. Of the patients initially included, almost 50% withdrew after randomization and the remainder showed moderate adherence to PR sessions. These two limitations reduced the power of this study to identify PR benefits. The characteristics of patients lost after initial evaluation did not differ significantly among the three randomized groups, indicating that loss to follow-up did not alter our final results.

In analyzing the factors related to non-adherence, we found that one of the primary health care centers was located far from the city center thus limiting accessibility to patients living far away. Low adherence during the second year of PR in our first cohort study resulted in a deviation from protocol, which was designed to evaluate the long-term (24-month) effect of PR maintenance. Patient withdrawal due to morbidity and mortality also had a negative influence on our final sample. Most studies analyzing similar rehabilitation programs in COPD patients have also shown considerable loss to follow-up, even higher than ours [15,41,42]. In addition, the PR provided in groups of patients probably made it more difficult to adapt to the timetable than if it were performed individually and, as such, influenced adherence to PR sessions.

## Conclusions

We found that patients with moderate COPD and low level of impairment did not show meaningful changes in QoL, exercise tolerance, pulmonary function or exacerbation after a one-year, community-based rehabilitation program. However, long-term improvements in the emotional, fatigue and mastery dimensions were identified. COPD is a disease in which most interventions, including pharmacological interventions, produce low levels of benefits that do not lead to quality of life improvements and a reduction in exacerbations. The selection of patients for community-based rehabilitation programs should be based on level of impairment and regular symptoms. The maintenance of benefits is highest in the group which completed the entire 12-month intervention although the long-term effects of PR programs is still unknown and more studies are needed to establish the optimum duration and nature of maintenance programs. These benefits are less evident in patients with moderate COPD.

## Competing interest

The authors’ declare that they have no competing interests.

## Authors’ contribution

MR, CL, AG and ME designed the study. ME and JR designed the questionnaires, coordinated trial field-work, acquisition of data and follow-up. IM and EZM elaborated the intervention, wrote the intervention leaflet and supervised physiotherapists. JR and ME did the statistical analysis and interpretation of data. MR, CL, ME, JR and AG wrote the paper. All authors provided critical revision for intellectual content, contributed to the writing of and approved the final paper.

## Pre-publication history

The pre-publication history for this paper can be accessed here:

http://www.biomedcentral.com/1471-2296/14/21/prepub
